# PRC2 is required for extensive reorganization of H3K27me3 during epigenetic reprogramming in mouse fetal germ cells

**DOI:** 10.1186/s13072-017-0113-9

**Published:** 2017-02-20

**Authors:** Lexie Prokopuk, Jessica M. Stringer, Kirsten Hogg, Kirstin D. Elgass, Patrick S. Western

**Affiliations:** 1 Department of Molecular and Translational Science, Centre for Genetic Diseases, Hudson Institute of Medical Research, Monash University, Clayton, VIC 3168 Australia; 2 Monash Micro Imaging, Monash University, Clayton, VIC 3800 Australia

**Keywords:** Epigenetics, Germ cells, Epigenetic reprogramming, Histone modifications, H3K27me3

## Abstract

**Background:**

Defining how epigenetic information is established in the germline during fetal development is key to understanding how epigenetic information is inherited and impacts on evolution and human health and disease.

**Results:**

Here, we show that Polycomb Repressive Complex 2 is transiently localized in the nucleus of mouse fetal germ cells, while DNA methylation is removed from the germline. This coincides with significant enrichment of trimethylated lysine 27 on histone 3 near the nuclear lamina that is dependent on activity of the essential PRC2 catalytic proteins, Enhancer of Zeste 1 and/or 2.

**Conclusions:**

Combined, these data reveal a role for Polycomb Repressive Complex 2 and trimethylated lysine 27 on histone 3 during germline epigenetic programming that we speculate is required to repress target sequences while DNA methylation is removed.

**Electronic supplementary material:**

The online version of this article (doi:10.1186/s13072-017-0113-9) contains supplementary material, which is available to authorized users.

## Background

The sperm and oocyte convey epigenetic information to the offspring that is essential for development and postnatal life. To ensure that appropriate epigenetic information is transmitted, germ cells undergo extensive reprogramming during fetal development. This allows existing parent-specific information to be reset and potential epigenetic errors to be removed from the germline. The most evident change during germline epigenetic reprogramming is the extensive removal of DNA methylation between embryonic day (E) 8.0 and E13.5, after which new DNA methylation is established in the male and female germlines in a sex-specific manner. Removal of germline DNA methylation occurs during two developmental stages. The first occurs during proliferation and migration of germ cells in the early embryo and involves reduction of DNA methylation levels from 70 to 30% [1]. This is followed by further reductions in DNA methylation as the germ cells enter the developing gonads and initiate sex-specific development. Combining this extensive epigenetic reprogramming results in global DNA methylation levels of ~14 and 7% in male and female germ cells at E13.5, respectively [1].

During this later period of reprogramming, DNA methylation is removed from imprint control regions (ICRs) of maternally and paternally imprinted genes, non-imprinted genes, intergenic and intronic sequences, and from the inactive X-chromosome in XX germ cells [2]. DNA methylation is also lost from many endogenous retroviral elements (ERVs), although relatively new ERVs are protected and retain relatively high levels of DNA methylation [1–6].

In addition to DNA demethylation, germline reprogramming involves extensive reorganization of histone modifications [7, 8]. At E8.5, dimethylated lysine 9 on histone 3 (H3K9me2) is exchanged for trimethylated lysine 27 on histone 3 (H3K27me3), which is maintained in the germ cell nucleus until at least E12.5 [9], although transient loss of H3K27me3 has been reported at E11.5 [7].

H3K27me3 is catalyzed by Polycomb Repressive Complex 2 (PRC2) and leads to epigenetic repression of target sequences. PRC2 is comprised of three core components: Enhancer of Zeste 1 or 2 (EZH1 and EZH2); Suppressor of Zeste 12 (SUZ12); and Embryonic Ectoderm Development (EED). While EZH2 is the primary catalytic component of PRC2 [10–12], EED is critical for physically binding H3K27me3 via five tandemly repeated WD motifs, thereby playing an essential role in PRC2 assembly [13, 14]. Inactivation of any one of the three core PRC2 protein subunits severely compromises the functional activity of the complex and results in the loss of H3K27me3. Complete loss of PRC2 function in mice is embryonic lethal [15–17], while low function (hypomorphic) mutations [18, 19] or tissue-specific deletion leads to growth and skeletal malformations [20], heart and immune cell defects [21] and increased susceptibility to tumorigenesis [22]. Conditional deletion of *Eed* in the male germline from around birth results in complete male infertility demonstrating an essential role for PRC2 in male germline development [23]. Although deletion of *Ezh2* in growing oocytes is compatible with normal fertility, offspring are born underweight, indicating that PRC2 acts as a maternal factor in oocytes [24]. However, PRC2 is required for germline development in *Drosophila* oocytes, where it regulates cell cycle progression during oocyte specification [25]. Moreover, PRC2 is required in the *Caenorhabditis elegans* germline to regulate the balance between H3K27me3 patterning catalyzed by MES 2, 3 and 6 (*C. elegans* PRC2), and MES4 which catalyzes H3K36me3 [26]. Despite these observations, the function of PRC2 in male and female in mammalian fetal germ cells remains unknown.

H3K27me3 is enriched at the promoter regions of many PRC2 target genes and plays an essential role in cell differentiation [27, 28]. In fetal germ cells, H3K27me3 is enriched at developmental genes [29–31] and is also enriched on nucleosomes that are retained at the promoters of developmental genes in mature sperm, indicating that PRC2 may regulate epigenetic information that is transmitted to offspring [32–34].

In this study, we identify a key period of transient PRC2 enrichment in gonadal germ cells as they undergo epigenetic reprogramming. Moreover, we demonstrate that PRC2 is required for significant transient enrichment of H3K27me3 near the nuclear lamina, specifically during the developmental period in which germline DNA methylation levels are at their lowest. We propose that PRC2 and H3K27me3 are required for epigenetic reprogramming in fetal germ cells and may provide a transient mechanism that protects certain sequences from aberrant expression during the period of reduced DNA methylation in the developing germline.

## Results

### H3K27me3 is highly enriched in fetal germ cells undergoing epigenetic reprogramming

**Fig. 1:**
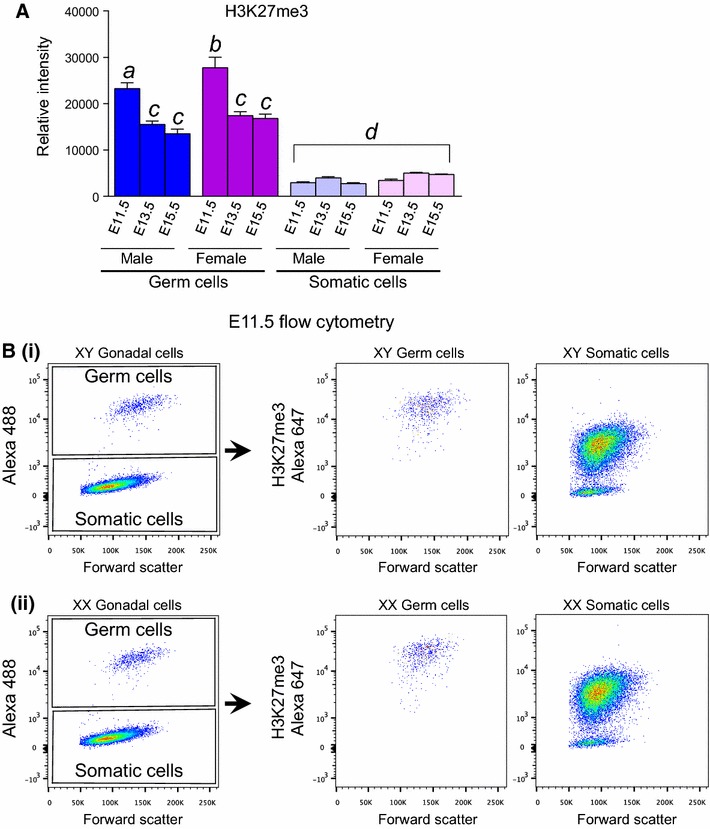
H3K27me3 is highly enriched in E11.5–E15.5 XX and XY germ cells. **A** Relative H3K27me3 antibody fluorescence intensities were measured using flow cytometry of dissociated and stained male (XY) and female (XX) fetal gonads at E11.5, E13.5 and E15.5. Germ cells were separated from somatic cells based on *Oct4*-eGFP expression (shown in **B**) and average ± SEM H3K27me3 levels measured in each population (*n* = 4). **B** Flow cytometric scatter plots showing gating of *Oct4*-eGFP positive germ cells and *Oct4*-eGFP negative somatic cells in XY (*i*) and XX (*ii*) E11.5 fetal gonads. Distribution of H3K27me3 intensities is shown for the germ cell (*middle plots*) and somatic cell populations (*right-hand plots*)

Initially, we used quantitative flow cytometric analyses to determine the overall cellular levels of H3K27me3 in E11.5, E13.5 and E15.5 male and female germ cells as they undergo epigenetic reprogramming. Germ cells were identified based on germ cell-specific expression of an *Oct4*-eGFP transgene [35–38] (Fig. 1A). At E11.5, XX and XY germ cells contained high levels of H3K27me3 (Fig. 1A, B), with no evidence in either sex of a germ cell population in which H3K27me3 was low or negative (Fig. 1B). Relative to E11.5 germ cells, H3K27me3 levels were moderately reduced in E13.5 germ cells of both sexes and then maintained until E15.5 (Fig. 1A). At all stages, germ cells contained substantially higher H3K27me3 levels than the gonadal somatic cells, which maintained relatively constant, lower levels of H3K27me3 (Fig. 1A). No H3K27me3 staining was detected in oocytes in which *Eed* had been conditionally deleted through expression of *Zp3*-*Cre*, confirming that the H3K27me3 antibody used specifically detects H3K27me3 (Additional file 1: Fig. S1).

### PRC2 is transiently expressed in fetal germ cells undergoing epigenetic reprogramming

**Fig. 2:**
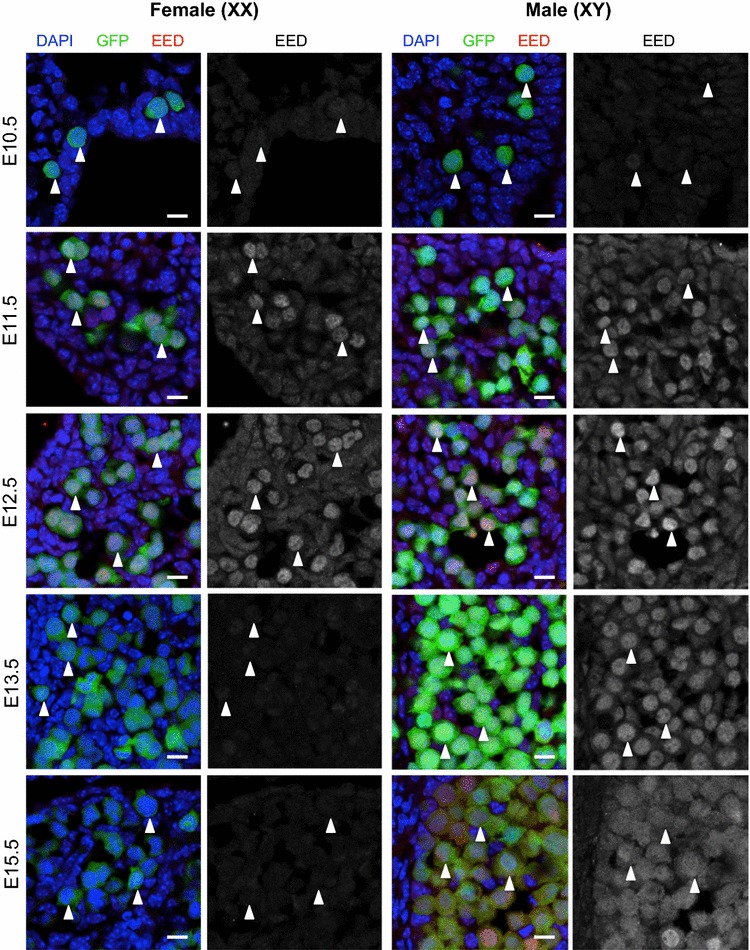
EED is enriched in E11.5–E12.5 male and female germ cells. Confocal images of EED immunofluorescence in sections of XX and XY E10.5–E11.5 bipotential gonad and E12.5–E15.5 developing ovaries and testes. *Left panels* are merged images: eGFP marking germ cells (*green*), EED (*red*) and DAPI nuclear stain (DNA; *blue*). *Right panels* are single-channel grayscale images showing EED staining. *White arrowheads* indicate *Oct4*-eGFP-expressing germ cells. Representative images chosen from 3 to 4 biological replicates for each time point. 10 μm *scale bars*

**Fig. 3:**
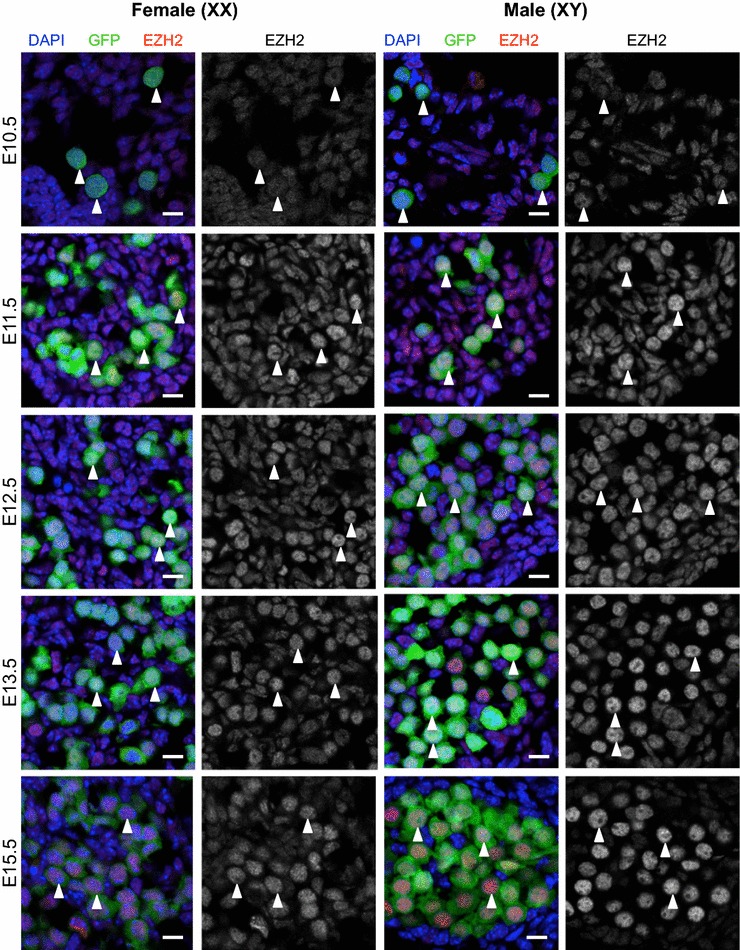
EZH2 protein is enriched in the nucleus of E11.5–E15.5 male and female germ cells. Confocal images of EZH2 immunofluorescence in sections of XX and XY E10.5–E11.5 bipotential gonad and E12.5–E15.5 developing ovaries and testes. *Left panels* are merged images: eGFP marking germ cells (*green*), EZH2 (*red*) and DAPI (DNA; *blue*). *Right panels* are single-channel grayscale images showing EZH2 staining. *White arrowheads* indicate *Oct4*-eGFP-expressing germ cells. Representative images chosen from 3 to 4 biological replicates for each time point. 10 μm *scale bars*

**Fig. 4:**
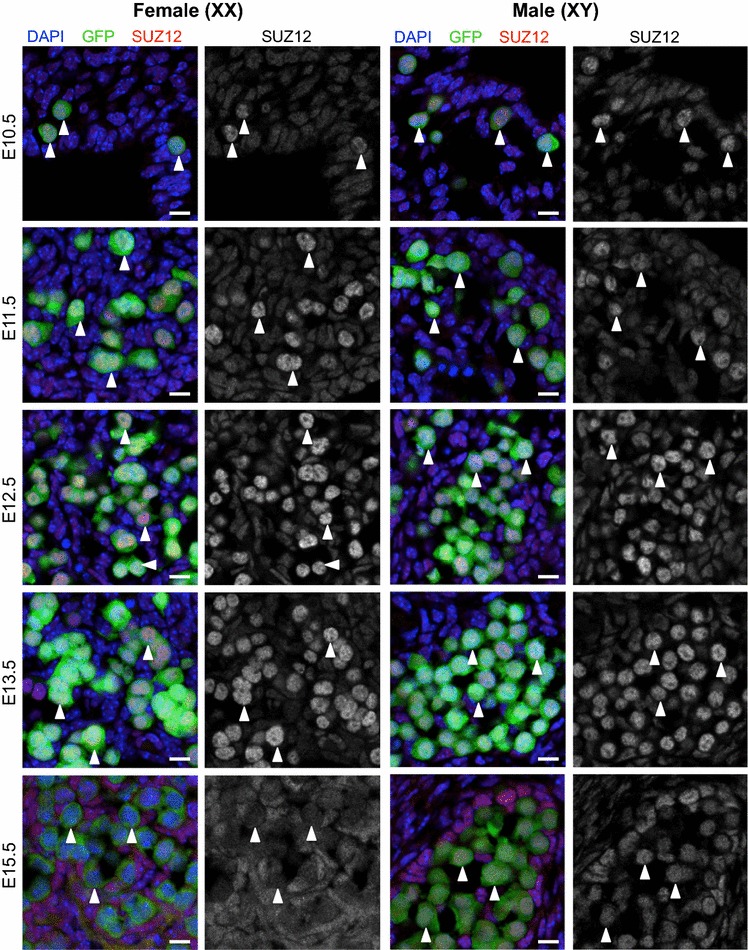
SUZ12 is enriched in the nucleus of E10.5–E13.5 male and female germ cells. Confocal images of SUZ12 immunofluorescence in sections of XX and XY E10.5–E11.5 bipotential gonad and E12.5–E15.5 developing ovaries and testes. *Left panels* are merged images: eGFP marking germ cells in *green*, SUZ12 (*red*) and DAPI (DNA; *blue*). *Right panels* are single-channel grayscale images showing SUZ12 staining. *White arrowheads* indicate *Oct4*-eGFP-expressing germ cells. Representative images chosen from 3 to 4 biological replicates for each time point. 10 μm *scale bars*

Next, we determined the temporal and spatial profile of the core PRC2 protein components EED, EZH2, SUZ12 and H3K27me3 in XX and XY fetal germ cells. Immunofluorescence (IF) was carried out on E10.5, E11.5, E12.5, E13.5 and E15.5 male and female gonads, coinciding with entry of germ cells into the developing gonad, epigenetic reprogramming in gonadal germ cells and early sex-specific germline development (Figs. 2, 3, 4). At E10.5, EED was not detected in either XX or XY germ cell (Fig. 2). EZH2 was detected with low staining intensity in the nucleus of some E10.5 XY germ cells but not XX germ cells (Fig. 3), and SUZ12 was detected with low staining intensity in the nucleus of both E10.5 XX and XY germ cells (Fig. 4). However, EED, EZH2 and SUZ12 were all readily detected in the nucleus of E11.5 and E12.5 XX and XY germ cells (Figs. 2, 3, 4). This pattern continued in E13.5 XX and XY germ cells (Figs. 2, 3, 4), with the exception of EED, which was not detected in E13.5 or E15.5 XX germ cells (Fig. 2). At E15.5, EED was weakly detected in the cytoplasm and nucleus of XY germ cells (Fig. 2). EZH2 was readily detected in E15.5 XX and XY germ cells (Fig. 3), while SUZ12 was only detected in XY germ cells at E15.5 (Fig. 4). We have summarized the relative staining intensity for each protein in E10.5–E15.5 XX and XY germ cells (Table 1).

**Table 1:** Relative staining intensities for PRC2 proteins in E10.5–E15.5 germ cells

	E10.5	E11.5	E12.5	E13.5	E15.5
Female XX					
EED	–	++	+++	–	–
EZH2	–	++	++	++	++
SUZ12	+	++	+++	+++	–
Male XY					
EED	–	++	+++	++	–/+ Cyto
EZH2	–/+	++	+++	+++	+++
SUZ12	+	++	+++	+++	++

### H3K27me3 is enriched near the nuclear lamina in fetal germ cells undergoing epigenetic reprogramming

**Fig. 5:**
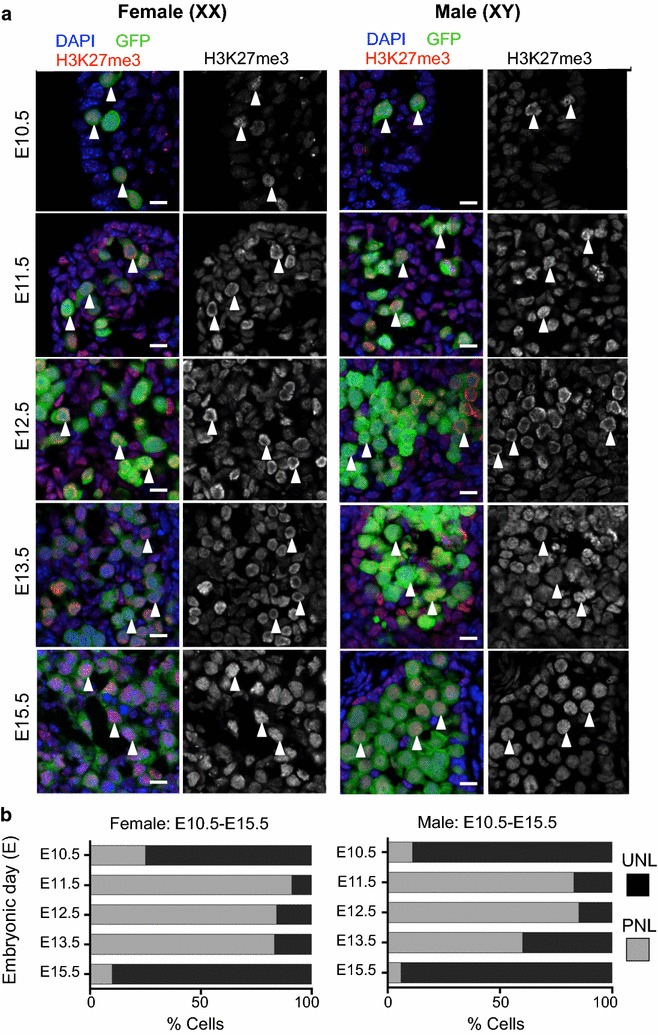
H3K27me3 is enriched in the nucleus of E10.5–E15.5 male and female germ cells. **a** Confocal images of H3K27me3 immunofluorescence in sections of XX and XY E10.5–E11.5 bipotential gonad and E12.5–E15.5 developing ovaries and testes. *Left panels* are merged images: eGFP marking germ cells in *green*, H3K27me3 (*red*) and DAPI (DNA; *blue*). *Right panels* are single-channel grayscale images showing H3K27me3 staining. *White arrowheads* indicate *Oct4*-eGFP-expressing germ cells. Representative images chosen from 3 to 4 biological replicates for each time point. 10 μm *scale bars*. **b** Quantification of H3K27me3 localization in E10.5–E15.5 male and female germ cells from wild-type fetal testis and ovary sections. Percentages of cells with uniform nuclear localization (UNL; *black bars*) and peripheral nuclear localization (PNL; *gray bars*) analyzed using ImageJ Cell Counter are shown in the stacked histogram. Data represent 3–6 biological replicates for each sex, at each stage (XX/XY cells counted: *n* = 16/19 at E10.5; *n* = 44/59 at E11.5; *n* = 196/214 at E12.5; *n* = 235/213 at E13.5 and *n* = 176/204 at E15.5)

Consistent with previous studies [7, 9, 39], H3K27me3 was detected relatively uniformly throughout the nucleus of E10.5 XX and XY germ cells (Fig. 5a). Although H3K27me3 was readily detected in E11.5 XX and XY germ cells, it was enriched close to the nuclear lamina, with little or no staining detected in the center of the nucleus (Fig. 5a). By E12.5, H3K27me3 staining was more obviously localized near the nuclear lamina in both XX and XY germ cells. H3K27me3 nuclear staining remained enriched close to the nuclear lamina in XX and XY germ cells at E13.5, but by E15.5 H3K27me3 was distributed throughout the nucleus in a pattern similar to that observed in E10.5 germ cells (Fig. 5a).

To quantify the proportion of germ cells that underwent a change from staining throughout the nucleus (uniform nuclear localization; UNL) to staining located toward the nuclear lamina (peripheral nuclear localization; PNL), we assessed the proportions of cells that had UNL and PNL at E10.5, E11.5, E12.5 and E15.5 in male and female gonads based on confocal images. At E10.5, H3K27me3 staining conformed to the UNL pattern in 75/90% of XX and XY germ cells (Fig. 5b). However, at E11.5 this pattern was significantly different, with 85/95% cells with H3K27me3 staining in the PNL pattern in XX and XY germ cells, and this pattern was maintained in E12.5 XX and XY germ cells. However, by E13.5 H3K27me3 staining returned to the UNL pattern in around 40% of XY germ cells but not in XX germ cells. By E15.5, 90/94% of XX and XY germ cells contained UNL, rather than PNL localization (Fig. 5b).

**Fig. 6:**
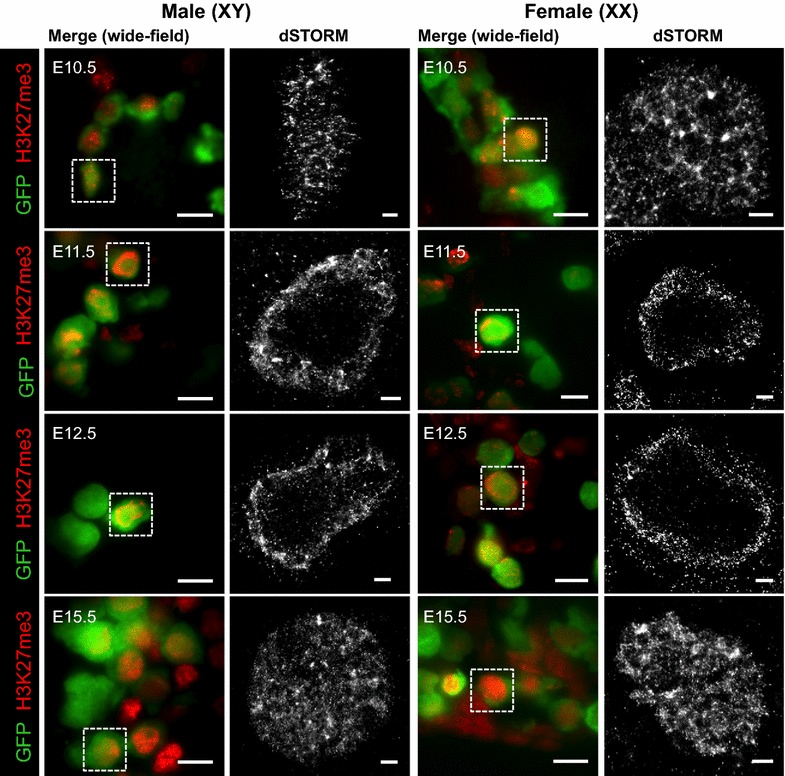
H3K27me3 is transiently relocated to the germ cell nuclear periphery in E11.5–E13.5 male and female germ cells. dSTORM super-resolution images in sections of XX and XY E10.5–E11.5 bipotential gonad and E12.5–E15.5 developing ovaries and testes. *Left panels* are merged wide-field (×160) images: eGFP marking germ cells (*green*), and H3K27me3 (*red*). *White dotted boxes* indicate super-resolved germ cell. 10 μm *scale bars*. *Right panels* dSTORM super-resolution of H3K27me3 antibody (*grayscale*). 1 μm *scale bars*. Representative images chosen from 3 to 8 super-resolved images in three biological replicates for each time point

To obtain greater resolution mapping of H3K27me3 localization in developing germ cells, we performed immunofluorescent super-resolution dSTORM imaging of H3K27me3 in E10.5, E11.5, E12.5 and E15.5 germ cells. Consistent with our observations using confocal imaging, H3K27me3 was distributed throughout the nucleus in both XX and XY germ cells at E10.5 (Fig. 6). This pattern changed dramatically at E11.5, with H3K27me3 located in a broadband around the periphery of the nucleus and by E12.5 H3K27me3 was typically detected in a relatively tight band around the nuclear periphery (Fig. 6). Remarkably, by E15.5 this pattern had completely reverted, and H3K27me3 was again localized throughout the nucleus in both XX and XY germ cells (Fig. 6). At this stage, XX and XY germ cells either have entered meiotic prophase or are mitotically arrested, respectively [36–38, 40–42].

**Fig. 7:**
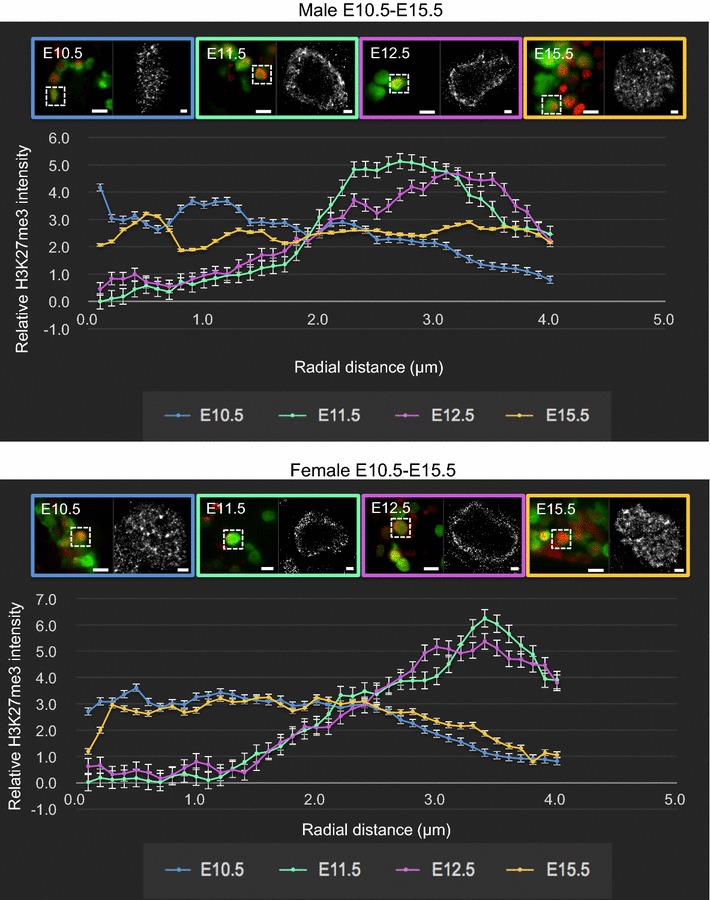
H3K27me3 is significantly enriched near the nuclear lamina in fetal germ cells undergoing epigenetic reprogramming. Radial histogram quantification of H3K27me3 localization of dSTORM super-resolution immunofluorescent images in wild-type male and female germ cells E10.5 (*blue*), E11.5 (*green*), E12.5 (*pink*) and E15.5 (*yellow*). *Left image* of each colored panel (×160) represents merged channels, *green* marking germ cells (eGFP), and H3K27me3 (*red*). 10 μm *scale bars*.* Right-hand* image of each* panel* shows dSTORM super-resolution images of germ cells (H3K27me3 in grayscale). 1 μm *scale bars*. Data represent 3–8 super-resolved images from three biological replicates. The *Y*-axis shows relative H3K27me3 intensity, and the *X*-axis shows radial distance from nucleus center (μm). Error bars ± SEM

To quantify the distribution of H3K27me3 located from the center to the outer edge of the nuclei of E10.5, E11.5, E12.5 and E15.5 germ cells, we developed an expanding circular quantification algorithm to measure blink intensity in the super-resolution images. Briefly, H3K27me3-derived pixels were counted in concentric circles increasing in 0.1 μm increments from the center of each nucleus to the nuclear lamina. At E10.5, H3K27me3 staining detected by dSTORM imaging was evenly distributed from the center of the nucleus to the nuclear lamina (Fig. 7). However, at E11.5 and E12.5 this pattern was strikingly different, with almost all enrichment for H3K27me3 staining located within 0–2 μm of the nuclear lamina in both sexes (Fig. 7). By E15.5, H3K27me3 was evenly distributed from the center of the nucleus to the nuclear lamina (Fig. 7). This confirmed a highly significant change in H3K27me3 from the UNL pattern at E10.5 to the PNL pattern during E11.5–E13.5 and again to the UNL pattern at E15.5.

### EZH1/2 is required for H3K27me3 enrichment near the nuclear lamina in fetal germ cells undergoing epigenetic reprogramming

Our examination of EED, EZH2 and SUZ12 protein expression revealed that all three core PRC2 proteins were present in XX and XY germ cells between E11.5 and E12.5, coincident with the enrichment of H3K27me3 near the nuclear lamina (Figs. 2, 3, 4; Table 1). To determine whether PRC2 function was required for H3K27me3 enrichment near the nuclear lamina during this period of germ cell development, we blocked EZH1/2 function using the highly specific EZH1/2 inhibitor GSK126 [43]. Initially, we validated the ability of GSK126 to block enrichment of H3K27me3 in germ cells. E11.5 whole gonad–mesonephros complexes were isolated from developing XX and XY embryos and cultured for 48 h on an organ culture membrane in medium containing increasing doses of GSK126 or vehicle control (DMSO). Flow cytometric analysis of GSK126 and control-treated gonads confirmed that GSK126 dose-dependently reduced H3K27me3 levels in germ cells in a 48-h period (Additional file 1: Fig. S2A). GSK126 was very well tolerated in these cultures, with even the highest dose having no effect on germ cell viability (Additional file 1: Fig. S2B). Moreover, we observed no impact on proliferation of either germ cells or somatic cells after 48-h treatment from E11.5 (Additional file 1: Fig. S2C, males shown). Similarly, in E12.5 fetal gonads cultured with 10 μm GSK126 for 72 h, germ cells entered mitotic arrest normally and somatic cell proliferation was normal, both in the Sertoli cell compartment and in the interstitial compartment (Additional file 1: Fig. S2D). This provided high confidence that GSK126 was well tolerated in fetal gonad cultures and demonstrated highly efficient, GSK126-dependent, depletion of H3K27me3 in germ cells within the 48- and 72-h culture periods.

**Fig. 8:**
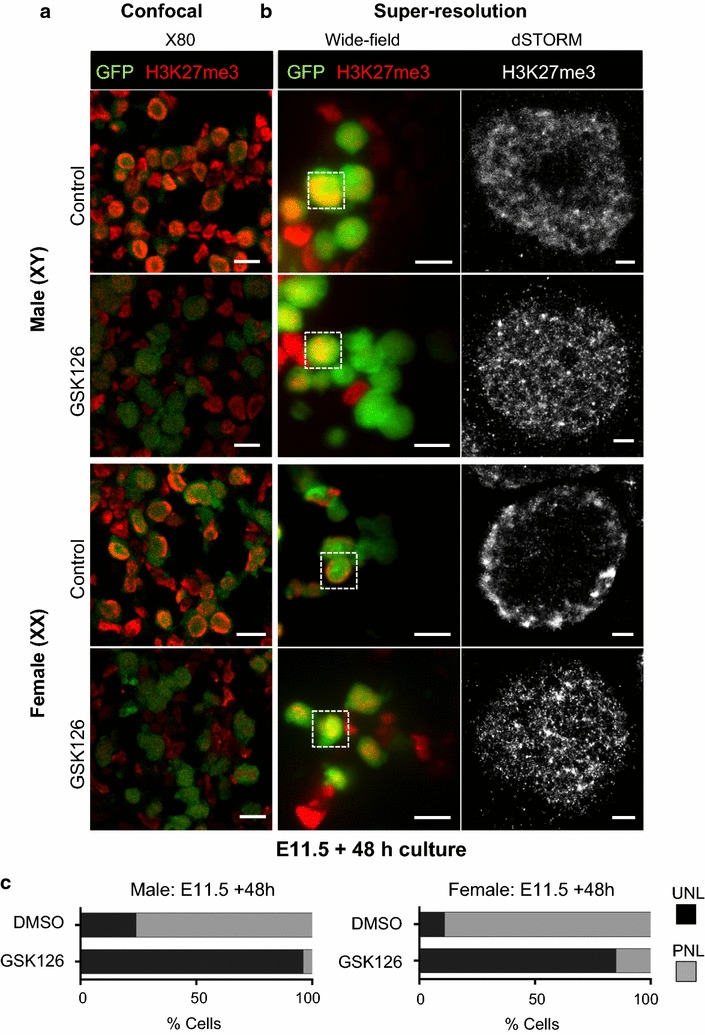
Transient enrichment of H3K27me3 near the nuclear lamina is lost when EZH1/2 is blocked. Confocal and dSTORM super-resolution images of immunofluorescence staining at E11.5 XX and XY gonads cultured for 48 h with either DMSO (control) or 10 μm GSK126. **a** Confocal images (×80) showing efficacy of H3K27me3 depletion by GSK126, eGFP (germ cells; *green*) and H3K27me3 (*red*). 10 μm *scale bars*. The reduction in H3K27me3 in GSK126-treated samples is quantified in Supp. Figure 3. **b** Super-resolution dSTORM images. *Left panels* show merged wide-field (×160) images: eGFP (germ cells: *green*) and H3K27me3 (*red*). *White dotted boxes* indicate super-resolved germ cells. 10 μm *scale bars*. *Right panels* show dSTORM super-resolution images of H3K27me3 (grayscale) control and GSK126-treated germ cells. 1 μm *scale bars*. Representative images chosen from 3 to 5 super-resolved images in three biological replicates for each time point. **c** Quantification of H3K27me3 localization in E11.5 XX and XY gonads cultured for 48 h with either DMSO (control) or 10 μm GSK126. ×80 confocal immunofluorescent images were analyzed by ImageJ Cell Counter. Percentages of cells with uniform nuclear localization (UNL; *black bars*) and peripheral nuclear localization (PNL; *gray bars*) are shown in the stacked histogram. Data represent 3–6 biological replicates for each sex, for each treatment group. (XX/XY cells counted: vehicle control *n* = 159/99; GSK126 *n* = 180/115)

**Fig. 9:**
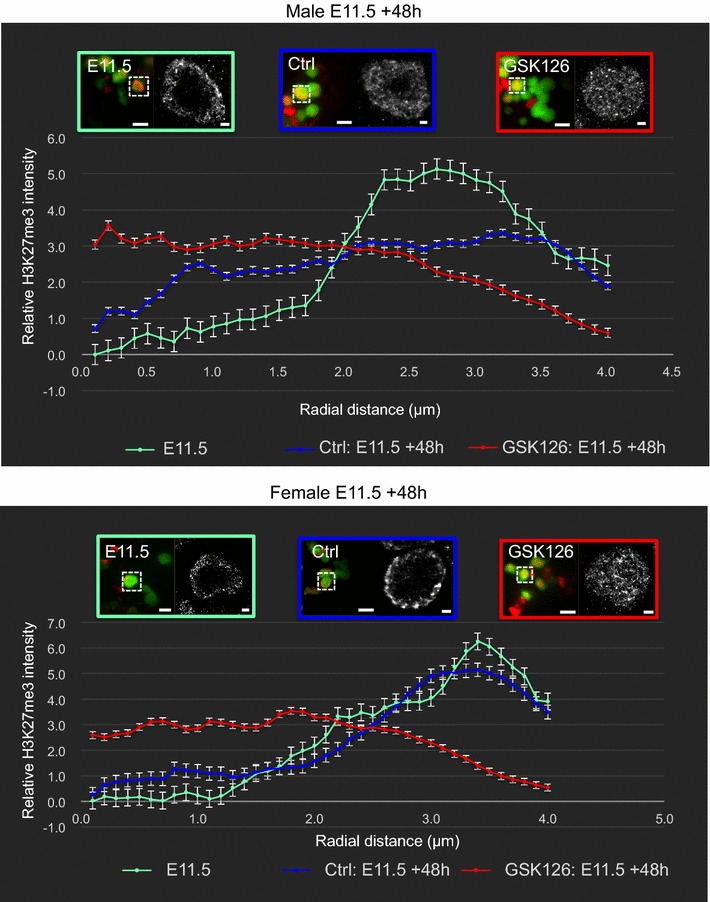
Blockage of EZH1/2 significantly reduces H3K27me3 enrichment near the nuclear lamina in fetal germ cells undergoing epigenetic reprogramming. Radial histogram quantification of dSTORM super-resolution immunofluorescent images of sections from E11.5 fetal testes and ovaries cultured for 48 h. E11.5 wild-type control is shown in *green*, E11.5 + 48 h vehicle control (DMSO) is shown in *blue*, and E11.5 + 48 h of GSK126 treatment (10 μm) shown in *red*. *Left image* of each* colored panel* (×160) represents merged channels, with* green* marking germ cells (eGFP) and H3K27me3 shown in the* red* channel. 10 μm *scale bars*. The* right-hand* image of each* panel* shows dSTORM super-resolution images of germ cells (H3K27me3 in grayscale). 1 μm *scale bars*. 3–8 super-resolved images from three biological replicates. *Y*-axis shows relative H3K27me3 intensity, and *X*-axis shows radial distance (μm) from the center of the nucleus.* Error bars* ± SEM

We next blocked EZH1/2 activity in E11.5 fetal gonads cultured with GSK126 for 48 h. Confocal imaging and analysis of H3K27me3 staining intensity using ImageJ demonstrated that H3K27me3 was reduced by 75 and 73% in germ cells of GSK126-treated male and female gonads compared to the germ cells of male and female control-treated gonads, respectively (*P* < 0.0001; Additional file 1: Fig. S3, Fig. 8a). Moreover, while H3K27me3 was detected in the periphery of the germ cell nucleus in control gonads, it was detected throughout the germ cell nucleus in GSK126-treated gonads (Fig. 8a), indicating a failure of this epigenetic mark to be enriched near the nuclear periphery when EZH1/2 function was blocked. Quantification of UNL and PNL staining revealed PNL staining in 89/75% of XX and XY germ cells in gonads treated with vehicle control, but only 15/4% in XX and XY germ cells of gonads treated with GSK126 for 48 h (Fig. 8c). To obtain a higher-resolution analysis of H3K27me3 localization in GSK126-treated germ cells, we performed super-resolution imaging on the same GSK126-treated and control gonads. In control-treated gonads, H3K27me3 staining was restricted to the periphery of the germ cell nucleus (Fig. 8b) in a pattern that was very similar to that observed in vivo in wild-type E12.5 germ cells (Fig. 6). However, in GSK126-treated gonads, remaining H3K27me3 was distributed throughout the germ cell nucleus (Fig. 8b) in a pattern that was indistinguishable from that observed in vivo at E10.5 and E15.5 (Fig. 6). Quantitative analysis of the localization of this staining demonstrated that the remaining H3K27me3 in germ cells of GSK126-treated gonads was evenly distributed throughout the nucleus and was not different from the pattern detected in untreated E10.5 and E15.5 germ cells. H3K27me3 was not enriched near the nuclear periphery of germ cells in GSK126-treated gonads, and its distribution was significantly different from the H3K27me3 enrichment observed near the nuclear lamina in normally developing E11.5 and E12.5 germ cells (Fig. 9). Interestingly in XY control gonads cultured for 48 h, H3K27me3 staining extended to ~3 μm from the nuclear periphery but was absent or low in the center of the nucleus. This pattern was comparable to the staining pattern observed by confocal microscopy in many E13.5 XY germ cells (Fig. 5b), indicating that H3K27me3 had already begun to relocate throughout the nucleus of control-treated gonads. We therefore examined XY gonads cultured for 24 h with control and GSK126 medium (Additional file 1: Fig. S4A). H3K27me3 levels were reduced by 57% in samples treated with GSK126 for 24 h compared to controls (*P* < 0.0001; Additional file 1: Fig. S4B). Quantification of UNL and PNL staining in confocal images revealed PNL staining in 85% of XY germ cells in gonads treated with vehicle control, but only 20% in XY germ cells of gonads treated with GSK126 for 24 h (Additional file 1: Fig. S4C). Super-resolution imaging of germ cells in these gonads revealed peripheral staining of H3K27me3 in control gonads and complete loss of peripheral staining in GSK126-treated gonads (Additional file 1: Fig. S5).

**Fig. 10:**
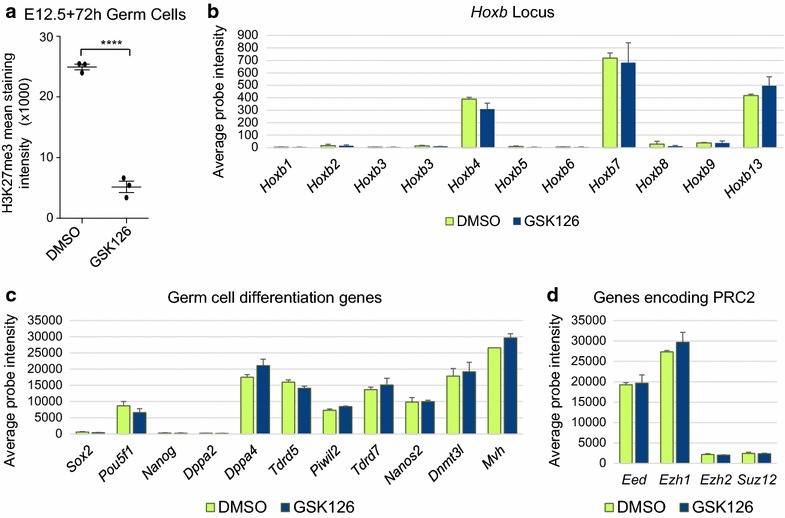
Gene expression profiles remained normal after H3K27me3 enrichment was reduced in fetal male germ cells. **a** Average H3K27me3 intensities were measured using flow analysis of FACS sorted E12.5 germ cells cultured for 72 h with a vehicle control (DMSO; *green*) or 10 μm GSK126 (*blue*). **b**–**d** Expression microarray relative probe signal intensities normalized to *Sdha*, *Canx* and *Mapk1* for genes expressed in E12.5 germ cells cultured for 72 h with DMSO or 10 μm GSK126: **b** *Hoxb* locus. **c** A selection of male germ cell development genes; *Sox2, Pou5f1, Nanog, Dppa2, Dppa4, Tdrd5, Piwil2, Tdrd7, Nanos2, Dnmt3l, Mvh*. **d** PRC2 genes; *Ezh1, Ezh2, Eed* and *Suz12*. Values ± SEM; *n* = 3 for each treatment

H3K27me3 enrichment is associated with the repression of many developmentally important genes, perhaps most notably the *Hox* genes. To identify genes for which depletion of H3K27me3 enrichment might alter expression, we treated male E12.5 fetal gonads for 72 h with GSK126. This covers a period during which many developmental genes are highly regulated in germ cells as they undergo early male germline differentiation. Initially, we confirmed efficient depletion of H3K27me3 using flow cytometric analysis of 10% of the gonadal cells collected for the GSK126 and vehicle control-treated cultures (Fig. 10a). This revealed an 80% decrease in H3K27me3 in germ cells isolated from GSK126-treated gonads compared to gonads treated with vehicle control. The remaining 90% of cells from the cultured gonads were subject to fluorescence-activated cell sorting (FACS), allowing purification of the germ cells and global expression analysis using microarrays. Surprisingly, analysis of global gene expression patterns using GeneSpring GX software revealed no significant differences in germ cell gene expression in gonads treated with GSK126 and vehicle control. In addition to global GeneSpring analysis, we used validated reference genes *Sdha*, *MapK1* and *Canx* [44] to normalize the raw array hybridization signals of known germ cell development, PRC2-regulated genes and genes encoding PRC2 proteins, and compared their relative expression in GSK126 and vehicle-treated samples. Consistent with the GeneSpring analysis, homeobox genes of the A, B, C and D clusters (Fig. 10b; *Hoxb* cluster shown), known male germ cell development genes (Fig. 10c), pluripotency genes (Fig. 10c), and PRC2 genes *Ezh1, Ezh2, Eed* and *Suz12* (Fig. 10d) were not significantly different in germ cells isolated from gonads treated with GSK126 or vehicle control for 72 h. In all cases, the expression of these genes was similar to what is expected for during normal germ cell development, with generally very low signals for the *Hox* genes and pluripotency genes *Nanog* and *Sox2*, moderate expression of *Oct4,* which is maintained in male germ cells at E15.5, high expression of *Dppa4, Piwil2, Tdrd5, Tdrd7, Nanos2, Dnmt3l* and *Mvh* which are upregulated in male germ cells by E15.5 and high expression of *Eed* and *Ezh1*, but relatively low expression of *Ezh2* and *Suz12.*

## Discussion

With the exception of DNA methylation, reprogramming of epigenetic information in the developing germline is poorly understood. Here, we demonstrate that the epigenetic modifier complex PRC2, which catalyzes H3K27 methylation, is transiently expressed in germ cells soon after they enter the gonad and undergo the final phase of DNA demethylation [3]. Coincident with increased PRC2 protein localization to the nucleus of germ cells, we observed substantial enrichment of H3K27me3 near the nuclear lamina in the gonadal germ cell population. This peripheral nuclear enrichment of H3K27me3 occurred during E11.5–E13.5, coinciding with the final phase of DNA demethylation and the lowest levels of DNA methylation in germ cells. By E15.5, H3K27me3 was again uniformly localized throughout nucleus in XX and XY germ cells. These findings demonstrate significantly three-dimensional reorganization of chromatin in the germ cell nucleus that is likely to be important for epigenetic reprogramming. In addition, we provide evidence that H3K27me3 enrichment near the nuclear lamina in E11.5–E13.5 germ cells is dependent on EZH1/2 activity, which comprise the essential catalytic proteins that mediate PRC2 activity. Combined, these data indicate that H3K27me3 mediates global reorganization of chromatin toward the nuclear lamina during the critical period during which germ cell DNA methylation levels are at their lowest. However, despite depletion of 80% of the H3K27me3 in male fetal germ cells, development of these cells was not altered within the 72-h culture window, and neither genes that mark male germline differentiation, nor known H3K27me3 targets, were altered.

A previous study reported reorganization of germ cell chromatin soon after germ cells enter the gonad [7]. This led to the proposal that H3K27me3 is removed and replaced in gonadal germ cells to ensure that epigenetic information is reset in the fetal germline [7]. However, another study concluded that H3K27me3 is maintained at relatively constant levels in gonadal germ cells, but fluctuates with cell cycle progression [9]. In the present study, we used flow cytometry to quantify H3K27me3 levels per cell and directly assess the variation in H3K27me3 levels in gonadal germ cells at E11.5, E13.5 and E15.5. In addition, we examined H3K27me3 in sections of gonads using both standard confocal imaging and super-resolution imaging at E10.5, E11.5, E12.5, E13.5 and E15.5. Using flow cytometry, we detected high levels of H3K27me3 in E11.5 germ cells, which moderately declined in E13.5 and E15.5 germ cells. H3K27me3 levels in germ cells were fourfold to sevenfold higher than those detected in the gonadal somatic cells at all stages analyzed. Consistent with this, confocal and super-resolution imaging revealed higher levels of H3K27me3 in germ cells than in somatic cells at all stages analyzed. Using these combined approaches, we found no evidence of extensive loss of H3K27me3 in either XX or XY germ cell after they entered the gonad. This is consistent with the conclusions of Kagiwada et al. [9], who also observed relatively constant levels of H3K27me3 in gonadal germ cells, although levels fluctuated with cell cycle. It remains possible that H3K27me3 is removed and replaced in a very short temporal window. However, based on our findings we conclude that widespread loss of H3K27me3 is unlikely to occur as part of epigenetic reprogramming in mouse germ cells in the few days after they enter the developing gonads.

Rather than loss of H3K27me3 from germ cells at E11.5, we observed substantial transient enrichment of H3K27me3 near the germ cell nuclear lamina. This persisted until E13.5, but by E15.5 H3K27me3 was again localized throughout the germ cell nucleus in both XX and XY germ cells. In male germ cells, the redistribution of H3K27me3 back through the nucleus at E15.5 coincided with the initiation of de novo DNA methylation in mitotically arrested male germ cells. However, H3K27me3 was also distributed throughout the nucleus by the time female germ cells exit the cell cycle and have entered meiotic prophase by E15.5 [37].

The redistribution of H3K27me3 toward the nuclear lamina in E11.5–E13.5 male and female germ cells is both striking and intriguing. One possibility is that H3K27me3 regulates specific gene expression programs in the male and female germlines during this time. However, our analysis of global gene expression patterns in which H3K27me3 was depleted by 80% revealed no differences in gene expression in the 72-h window analyzed. Markers of male germline development were expressed normally, and there was no evidence that known targets of PRC2/H3K27me3 were de-repressed. This was surprising as previous studies have identified genes that are enriched for H3K27me3 in male and female germ cells during this period, suggesting that PRC2-mediated H3K27me3 enrichment might substantially regulate sex-specific germ cell development [29–31]. One caveat for the current study is that around 20% of the global H3K27me3 levels remained in germ cells after drug treatment, and this may be sufficient to maintain normal regulation of gene expression. However, dSTORM imaging clearly demonstrated that the H3K27me3 that remained in GSK126-treated cells was no longer localized toward the nuclear lamina, demonstrating that at least this function was compromised in the drug-treated gonads. Moreover, as we observed similar enrichment of H3K27me3 near the nuclear lamina in both sexes, and inhibition of EZH1/2 and depletion of H3K27me3 affected this equally in both sexes, it is unlikely that the global reorganization of H3K27me3 we observed can explain the regulation of sex-specific programs. It is more likely that H3K27me3 mediates a repressive activity that affects sequences common to both male and female germ cells.

Based on the current data, the most plausible explanation for the enrichment of H3K27me3 near the nuclear periphery is that H3K27me3-enriched chromatin is relocalized to the nuclear lamina in order to achieve appropriate transcriptional repression. Although it remains possible that H3K27me3 is removed from the center of the nucleus between E10.5 and E11.5 and re-established in the center of the nucleus between E13.5 and E15.5, we consider this unlikely for two reasons. Firstly, blocking EZH1/2 which catalyzes H3K27me3 prevented enrichment of H3K27me3 at the germ cell nuclear periphery in gonads cultured from E11.5 for 24 or 48 h. Secondly, studies in other cell systems provide substantial evidence that localization of chromatin to the nuclear periphery is associated with transcriptional repression [45, 46].

Indeed, it is highly plausible that transcriptional repression plays an essential role in maintaining genomic integrity during epigenetic reprogramming. DNA methylation is at its lowest level soon after germ cells enter the gonad. This is considered to involve a period during which germ cells may be vulnerable to aberrant expression of sequences such as ERVs that would otherwise contain DNA methylation and be repressed [2, 6, 47–49]. Previous studies have demonstrated enrichment of H3K27me3 at ERVs including young LTR and LINE elements, and at intergenic regions, introns and ICRs in male and female gonadal germ cells undergoing epigenetic reprogramming [6, 31]. Although repression of ERVs is in part achieved by SETDB1 through its catalysis of H3K9me3 in E13.5 fetal germ cells, this activity does not account for the repression of all ERVs, indicating that other mechanisms also repress ERVs during this period [6]. Moreover, recent work has demonstrated a role for H3K27me3 in ERV repression in embryonic stem cells in which DNA methylation levels are reduced by treatment with 5-azacytidine and vitamin C [50].

Interestingly, although H3K27me3 was enriched near the nuclear lamina in germ cells undergoing reprogramming, EZH2, EED and SUZ12 staining was consistently detected throughout the nucleus. Yet blocking EZH2 activity prevented enrichment of the remaining H3K27me3 near the nuclear lamina. This implies that although PRC2 is required for catalyzing H3K27me3 in the nucleus at this stage, the complex is not relocated toward the nuclear lamina with H3K27me3-enriched chromatin. One possible explanation is that relocalization of H3K27me3 may depend on an additional mechanism(s) in the germ cell nucleus.

In this study, we demonstrate PRC2-dependent enrichment of H3K27me3 near the nuclear lamina when DNA methylation is at its lowest level in germ cells, but no change in expression of protein coding genes, with the caveat that EZH1/2 was blocked and H3K27me3 depleted in a limited 72-h window. However, EZH1/2-dependent reorganization of H3K27me3 in both sexes suggests that H3K27me3 plays roles other than repressing coding genes during this developmental period. One possibility may be that PRC2 and H3K27me3 play a transient role in repressing ERVs during epigenetic reprogramming of the germline through a mechanism that involves their relocalization to the nuclear periphery and sequestration into repressive chromatin structures. However, further work is required to determine whether this is the case.

## Conclusions

We have identified substantial nuclear reorganization of the repressive histone modification H3K27me3 in germ cells as they undergo epigenetic reprogramming and reach their lowest DNA methylation levels. This occurs during a transient period of PRC2 enrichment in germ cells of both sexes and is dependent on EZH2 activity, the enzyme that catalyzes H3K27 methylation. Determining the repressive mechanisms that regulate germline epigenetic state during germline reprogramming is essential for understanding how altered epigenetic function in the germline contributes to inherited disease. It is therefore imperative that further work seeks to understand the mechanisms that regulate epigenetic and genetic integrity in the germline prior to fertilization.

## Methods

### Mouse strains, animal housing, breeding and ethics

Mice were housed at Monash Medical Centre Animal Facility using a 12-h light–dark cycle. Food and water were available ad libitum, and room temperature was 21–23 °C with controlled humidity. With the exception of breeding pairs and neonatal mice (up to three weeks old), males and females were weaned at 21 days and kept in cages of up to five individuals. All animal work was undertaken in accordance with Monash University Animal Ethics Committee (AEC) approvals. Temporal and spatial profiling of PRC2 components and H3K27me3 was carried out on embryos collected from *Oct4* [*Pou5f1*]-eGFP on 129svJ background males crossed with Swiss females (obtained from Monash Animal Services). Females were checked for vaginal plugs daily, and detection of a plug was noted as E0.5. Embryos E12.5 or older were sexed by the presence (male) or absence (female) of testis cords in gonads. Embryos younger than E12.5 were sexed using PCR as described [51].

### Flow cytometry

Gonad collection, dissociation, fixation, antibody staining and flow cytometry are as previously described [52]. Gonad samples stained with rabbit IgG control antibody were used as negative controls to set flow cytometry gates for H3K27me3 intensity, and mesonephros samples were used as a germ cell negative control to gate for eGFP. Cell proliferation was assessed by dosing cultured gonads with 20 μm 5-ethynyl-2 deoxyuridine (EdU) for the final 2 h of culture prior to tissue collection, dissociation and fixation. Cell cycle analysis was carried out as described [53] with germ cells identified by their expression of mouse vasa homologue (MVH) and somatic cells identified by anti-Mullerian hormone (AMH) staining. Cells were stained with 20 μg/ml propidium iodide, allowing quantitation of cellular DNA content. Proliferation was measured by gating EdU-positive cells against propidium iodide to identify cells actively in S-phase. Cells in G1 and G2/M were identified by their DNA content and the absence of S-phase activity. All flow cytometry was performed on a FACS Canto instrument and data analyzed in FlowJo and GraphPad Prism. For all analyses, 3–9 biological replicates were analyzed and statistical significance determined using one-way ANOVA with Tukey’s multiple comparison or *t* test as appropriate. *P* values <0.05 were considered significant.

### Tissue fixation and embedding

Gonads were fixed in 4% paraformaldehyde (PFA) in PBS for IF and confocal imaging, or 2% PFA plus 0.02% glutaraldehyde (GA) in PBS (dSTORM super-resolution microscopy) for 20–90 min according to embryo age (Additional file 1: Table S1). Samples were then washed twice in PBS and left in 30% sucrose in PBS overnight at 4 °C. Samples were then placed in disposable cryostat molds (Sakura Finetek, 4565) filled with OCT (Sakura Finetek, 4583) and frozen in dry ice. Blocks were and stored at −80 °C.

### Immunofluorescence

Eight-micron sections were cut from OCT-embedded gonads fixed in 4% PFA, mounted on SuperFrost Plus slides and dried for 5 min before immersing in 1× PBS. Sections were then permeabilized by incubation in 1% Triton × 100 (Sigma, T8787) in PBS for 10 min at room temperature (RT). Slides were washed three times for 5 min each in PBS. Sections were blocked in PBS containing 5% BSA (Sigma, A9647) and 10% donkey serum (Sigma, D9663) and incubated for 45 min at RT. Blocking solution was replaced by PBS containing 1%BSA and appropriately diluted primary antibodies (Additional file 1: Table S2) and incubated for 1 h at RT. Slides were washed three times for 5 min in PBS and secondary antibodies diluted in 1% BSA in 1 x PBS according to antibody dilutions outlined in Additional file 1: Table S3. Secondary antibody incubation was carried out in a dark box for 1 h at RT. Slides were washed three times in PBS (5 min each wash) and mounted in ProLong Gold^**®**^ containing DAPI (Life Technologies, P36931) and left in a dark box over night to dry. For control slides, only a secondary antibody was applied. Confocal images were taken as single optical sections using a Nikon^**®**^ C1 inverted confocal microscope. All pictures were taken at 80×, using a 40× oil immersion lens. Nuclear localization of H3K27me3 was assessed in 3–6 biological replicates per embryonic stage. Each germ cell was visually assessed for UNL or PNL staining based on the presence of staining throughout the nucleus or only around the nuclear periphery, respectively. Cell counts were recorded using ImageJ Plugin Cell Counter. Data were expressed as the percentage of cells with UNL and PNL staining and analyzed for statistically significant differences between stages using one-way ANOVA with Tukey’s multiple comparison test or differences between treatments using Student’s *t* test with *P* ≤ 0.05 considered significant. H3K27me3 mean intensity was measured in three biological replicates for each culture period (E11.5 +48 h male and females, >119 cells analyzed; E11.5 +24 h males, >68 cells analyzed). ImageJ ROI manager was used to calculate the mean intensity for H3K27me3, and unpaired *t* tests were used to statistically analyze treatments groups, with *P* ≤ 0.05 considered significant.

### dSTORM super-resolution imaging

Two-micron sections were cut from OCT-embedded gonads fixed in 2% PFA and 0.02% GA (Additional file 1: Table S1) and mounted on poly-l-lysine (Sigma) coated #1.5 Coverslips (Grale HDS) enclosed within 8-well sticky chambers (Ibidi, 80828) and stained.

Super-resolution images were recorded using a custom-built (Monash Micro Imaging) dSTORM instrument based on an Olympus IX-71 microscope equipped with Plan ApoChromat oil immersion 100× 1.4NA objective (Olympus part #N1480900), 1.6× magnification changer, Toptica 488 nm laser (200 mW), Gem 561 nm laser (500 mW), and Oxxius 637 nm laser (150 mW), suitable Olympus fluorescence filter cubes and Andor iXon Ultra 897 High Speed EMCCD camera with single-photon sensitivity for single-molecule detection. The final excitation steering mirror and beam expansion lenses were mounted on a translation stage for free adjustment of the total internal reflection fluorescence (TIRF) angle. The system was operated at a TIRF angle appropriate for the respective sample to concentrate excitation power and reduce background fluorescence. Samples were mounted on a manual *x*,*y* translation stage to minimize sample drift.

Alexa Fluor^®^ 647 super-resolution imaging was performed in imaging buffer at pH8.0 containing 50 mM MEA and a glucose scavenging system (10% Glucose, 40 μg/ml catalase, 0.5 mg/ml glucose oxidase) in PBS. The sample was illuminated continuously with 70 mW 637 nm laser power at the appropriate TIRF angle. After an initial pumping period of <30 s to drive dyes into the dark state, single-molecule blinking time series were acquired for 10,000 frames at a camera EM gain of 50 and exposure time of 20 ms.

The acquired data were reconstructed to super-resolved images of 10-nm pixel size using the open-source software rapidSTORM version 3.3.1. Blinks with a local signal-to-noise ratio (SNR) <80 were discarded. Images were first color coded for temporal appearance of blinks to detect sample drift, then (if necessary) corrected for drift using the linear drift correction available in rapidSTORM and exported as 8-bit grayscale images.

### Analysis of H3K27me3 staining in dSTORM super-resolution images

Radial histogram analysis was carried out for all representative super-resolution images for each embryonic time point and treatment group. This involved the use of an algorithm (macro was installed and analyzed on ImageJ) to measure staining intensity of H3K27me3, beginning at the center of the cell nucleus out to the nuclear periphery. H3K27me3 intensity was measured in ever-increasing concentric circles (radial diameter) expanding in at 0.1 μm increments from the center of the nucleus until the nuclear lamina was reached. Values on each radial diameter line were collated and averaged to provide values ± standard error of the mean across 3–6 biological replicates. Data were processed and graphed in Microsoft Excel.

### Organ culture

All culture reagents were purchased from Life Technologies unless otherwise stated. Embryos were collected at E11.5 from Swiss females mated to 129T2svJ *Oct4*-eGFP transgenic males. Gonad plus mesonephros was cultured on 30-mm organotypic cell culture inserts (Merck Millipore; PICM03050) in 1200-μl culture media (250 μm sodium pyruvate, 15 mM Hepes, 1X nonessential amino acids (Life Technologies, 11140), 1 mg/ml N-acetylcysteine (Sigma, A9165), 55 μm β-mercaptoethanol (Life Technologies, 21985) and 10% FCS in DMEM/F12 with Glutamax (Life Technologies, 10565) containing either DMSO (vehicle control) or 10 μm GSK126 (EZH1/2 inhibitor; SelleckChem, S7061). Media preparations also contained 1× penicillin/streptomycin (Life Technologies, 15070). Gonads were randomly allocated to each culture treatment condition and cultured for 48 h in 37 °C/5% CO_2_ conditions. Culture media was refreshed daily. Gonads were processed for flow cytometry, FACS, IF and dSTORM super-resolution imaging.

### FACS and cell viability

Four to six cultured gonads were collected, pooled together and dissociated with 0.25% trypsin as previously described. The reaction was stopped by adding 500 μl of culture media containing 10% fetal calf serum, and cells were collected by centrifugation. Cells were resuspended in 300 μl culture media containing 10% fetal calf serum and propidium iodide added to a final concentration of 2 μg/ml. Cells were run on an Influx 2 cell sorter. Non-viable cells were quantified and excluded as a percentage of the whole single cell population based on propidium iodide staining. *Oct4*-*e*GFP positive (germ) and negative (somatic) cells separated based on eGFP fluorescence and collected in separate tubes. Cells were collected by centrifugation and snap frozen for RNA extraction.

### RNA preparation and expression microarray analysis

RNA was extracted from FACS-purified cells (~20,000 cells per sample, *n* = 3 for vehicle and GSK126 treatments, respectively) using TRIzol reagent, treated with Turbo DNAse 1 (Ambion) according to the manufacturer’s instructions and resuspended in RNAase-free water. RNA quantity and quality were assessed using a Qubit instrument and a Bioanalyzer. Total RNA (100 ng/sample) was labeled using Agilent One Colour Low Input Quick Amp Labelling Kit v.6.6, and dye incorporation assessed using a NanoDrop ND-1000 Spectrophotometer. In total, 600 ng of Cy3-labeled cRNA (specific activity >6 pmol Cy3/μg) was fragmented at 60 °C according to the Agilent protocol and hybridized to Agilent SurePrint G3 Mouse Gene Expression 8 × 60K Arrays for 17 h at 67 °C according to manufacturer’s instructions. Arrays were washed and immediately scanned on an Agilent C, DNA microarray scanner using one-color scan settings for 8 × 60K arrays. The scanned images were analyzed with Feature Extraction Software 11.0.1.1 (Agilent) using default parameters (protocol GE1-1100_Jul11 and Grid: 028005_D_F_20120201) to obtain background subtracted and spatially detrended Processed Signal intensities. Data were analyzed using GeneSpring GX software using the proprietary analysis pathway. In addition, raw signal intensities for a selection of genes were independently normalized against validated reference genes for fetal germ cells using intensities across all probes for *Sdha*, *Canx* and *MapK1* and the data expressed as normalized signal intensities.

## Additional file

**Additional file 1.** PRC2 is required for extensive reorganization of H3K27me3 during epigenetic reprogramming in mouse fetal germ cells.

## Abbreviations

AMH: anti-Mullerian hormone (also known as Mullerian inhibitory substance)
Cyto: cytoplasm
EED: Embryonic Ectoderm Development
E: embryonic day
eGFP: enhanced GFP
ERVs: endogenous retroviral elements
ERV1: Class I
ERVK: Class II
EZH1: Enhancer of Zeste 1
EZH2: Enhancer of Zeste 2
FACS: fluorescence-activated cell sorting
GSK126: EZH1/2 inhibitor
H3K9me2: dimethylated lysine 9 on histone 2
H3K9me3: trimethylated lysine 9 on histone 3
H3K27me3: trimethylated lysine 27 on histone 3
IAPs: intracisternal-A-particles
LINE: long interspersed nuclear elements
LINE1: LINE subgroup 1
MVH: mouse vasa homologue (also known as DDX4)
PNL: peripheral nuclear localization
PRC2: Polycomb Repressive Complex 2
SETDB1: SET domain, bifurcated 1
SINE: short interspersed elements
SUZ12: Suppressor of Zeste 12
TEs: transposable elements
UNL: uniform nuclear localization
