# Supplementary material for: PRC2 is required for extensive reorganization of H3K27me3 during epigenetic reprogramming in mouse fetal germ cells
Source: Epigenetics Chromatin. 2017 Feb 20;10:7. doi: 10.1186/s13072-017-0113-9 (PMC5319108; doi:10.1186/s13072-017-0113-9)
Supplement: Supplementary file 1 — Additional file 1. PRC2 is required for extensive reorganization of H3K27me3 during epigenetic reprogramming in mouse fetal germ cells. [file 13072_2017_113_MOESM1_ESM.pdf]

Additional file 1:

PRC2 is required for extensive reorganization of H3K27me3 during epigenetic reprogramming in mouse fetal germ cells

Lexie Prokopuk[1], Jessica M Stringer[1], Kirsten Hogg[1], Kirstin D Elgass[2], Patrick S Western[1#]

[1] Centre for Genetic Diseases, Hudson Institute of Medical Research and Department of Molecular and Translational Science, Monash University, Clayton, Victoria, Australia 3168.

[2] Monash Micro Imaging, Monash University, Clayton, Victoria, Australia 3800.

[#]Corresponding author

patrick.western@hudson.org.au

Tel: +61 3 8572 2673

Supplementary Figures and Tables:

Fig. S1: H3K27me3 and EED immunofluorescent staining is lost in *Eed* deleted mouse oocytes: validation of H3K27me3 and EED antibody specificity. Confocal images of H3K27me3 and EED immunofluorescence in sections of adult mouse $Eed^{wt/wt}$ (top images) and $Eed^{del/del}$ (bottom images) ovaries from *Eed-Zp3Cre* females. Left panels are merged images: H3K27me3 or EED (red) and DAPI nuclear stain (blue). Rights panels are single channel greyscale images showing A. H3K27me3 or B. EED staining. White dotted circles outline the oocyte nucleus. Representative images chosen from 3-4 biological replicates. 10μm scale bars.

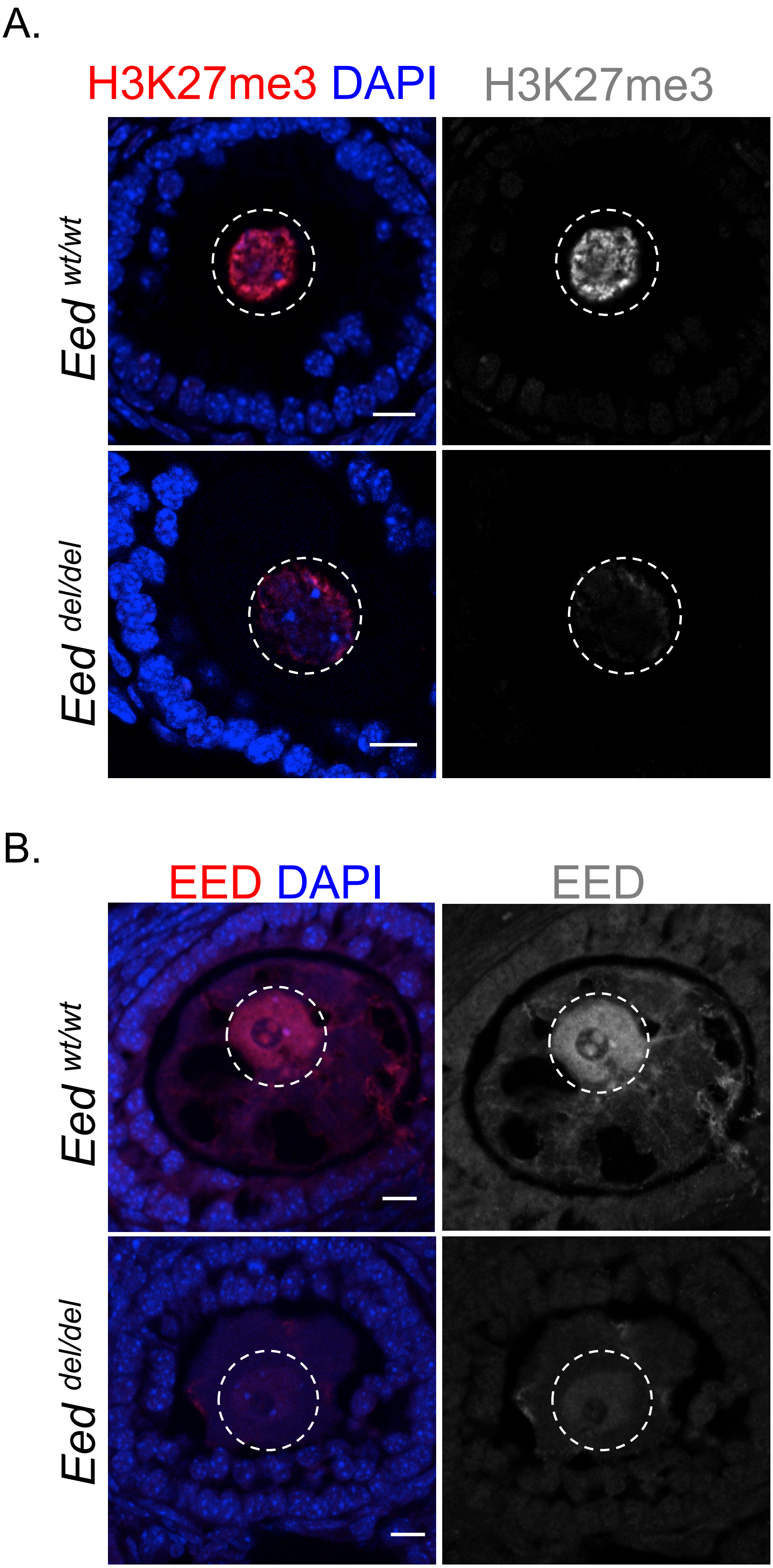

Prokopuk *et al* Additional file 1: Fig. S1

Fig. S2: GSK126 treatment robustly depletes H3K27me3 in germ cells without affecting germ cell viability or proliferation. A. Flow cytometric analysis of H3K27me3 average mean staining intensity in XX (purple) and XY (blue) germ cells from E11.5 gonads cultured for 48 hours with vehicle control (DMSO) (left) or 1µm, 5 µm and 10 µm GSK126. B. Proportion of viable germ cells (XX and XY) after E11.5 + 48h gonad culture period with GSK126 (10 µm; blue) or vehicle control (DMSO; green) measured using propidium iodide staining and fluorescence activated cell sorting for viable germ cells. Germ cells were identified by *Oct4*-eGFP fluorescence. C. Flow cytometric cell cycle analysis of isolated germ (left) and somatic cells (right) from E11.5 XY gonads cultured for 48h with GSK126 (10 µm; blue) or vehicle control (DMSO; green). Data represents average ± SEM of n=3-5 biological replicates per treatment group. D. Flow cytometric cell cycle analysis of germ (left) and somatic cells (right) from E12.5 XY gonads cultured for 72h with GSK126 (10 µm; blue) or vehicle control (DMSO; green). Data represents average ± SEM of n=3 samples per treatment group.

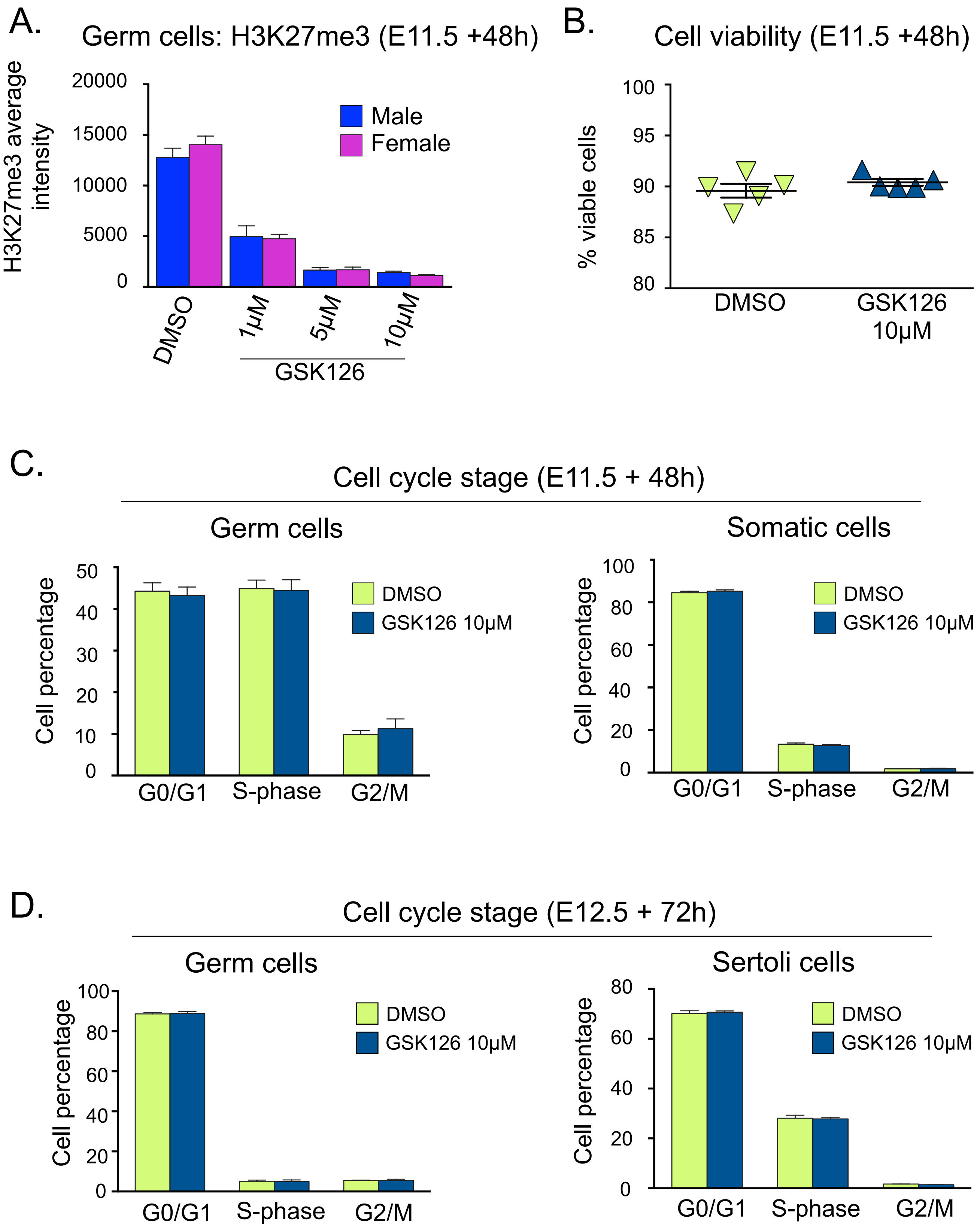

Prokopuk *et al* Additional file 1: Fig. S2

Fig. S3: Treatment of male and female gonads with GSK126 significantly reduced H3K27me3 levels in germ cells. A. ImageJ analysis of the H3K27me3 immunofluorescence staining intensity in germ cells of E11.5 XY (male) gonads treated with DMSO (control) or 10uM GSK126 for 48h. (Student's *t*-test: $P<0.0001$; n=119 and 186 germ cells and average intensities = 2285158 and 561793, respectively). B. ImageJ analysis of the H3K27me3 immunofluorescence staining intensity in germ cells of E11.5 XX (female) gonads treated with DMSO (control) or 10uM GSK126 for 48h. (Student's t-test: $P<0.0001$; n=162 and 206 germ cells and average intensities = 2166811 and 576732, respectively).

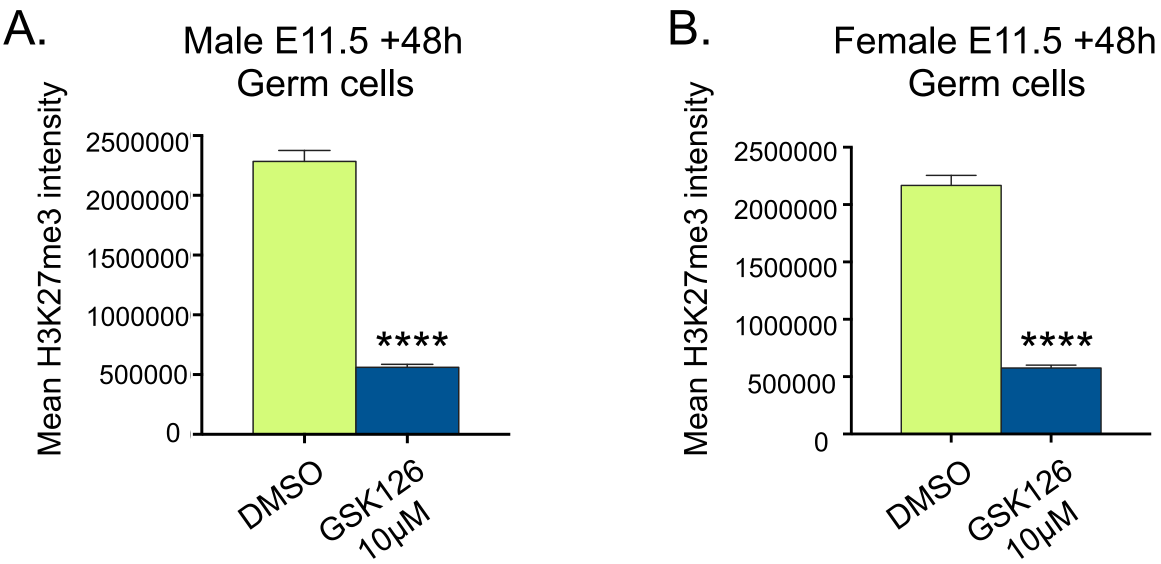

Prokopuk *et al* Additional file 1: Fig. S3

Fig. S4: Transient enrichment of H3K27me3 near the nuclear lamina is lost when EZH1/2 is blocked. A. Confocal and dSTORM super-resolution images of immunofluorescence staining at E11.5 XX and XY gonads cultured for 24h with either DMSO (control) or 10μM GSK126. Confocal images (left images; 80x) showing efficacy of H3K27me3 depletion by GSK126. Germ cells are shown in green (eGFP) and H3K27me3 shown in red. 10μm scale bars. Wide field (middle images) show cells imaged using dSTORM and dSTORM (right images) show H3K27me3 localisation. B. ImageJ analysis of the H3K27me3 immunofluorescence staining intensity in germ cells of E11.5 XY (male) gonads treated with DMSO (control) or 10uM GSK126 for 24h (Student's t-test: $P<0.0001$; n=86 and 68 and average intensities = 2040347 and 869654, respectively). C. Quantification of H3K27me3 localization in germ cells of E11.5 XY gonads cultured for 24h with either DMSO (control) or 10μM GSK126. 80X confocal immunofluorescent images were analyzed using ImageJ Cell Counter. Percentages of cells with uniform nuclear staining (UNL; black bars) and peripheral nuclear staining (PNL; grey bars) are shown in the stacked histogram. Data represents 3 biological replicates for each treatment group. (Germ cells counted: vehicle control n=141; GSK126 n=70).

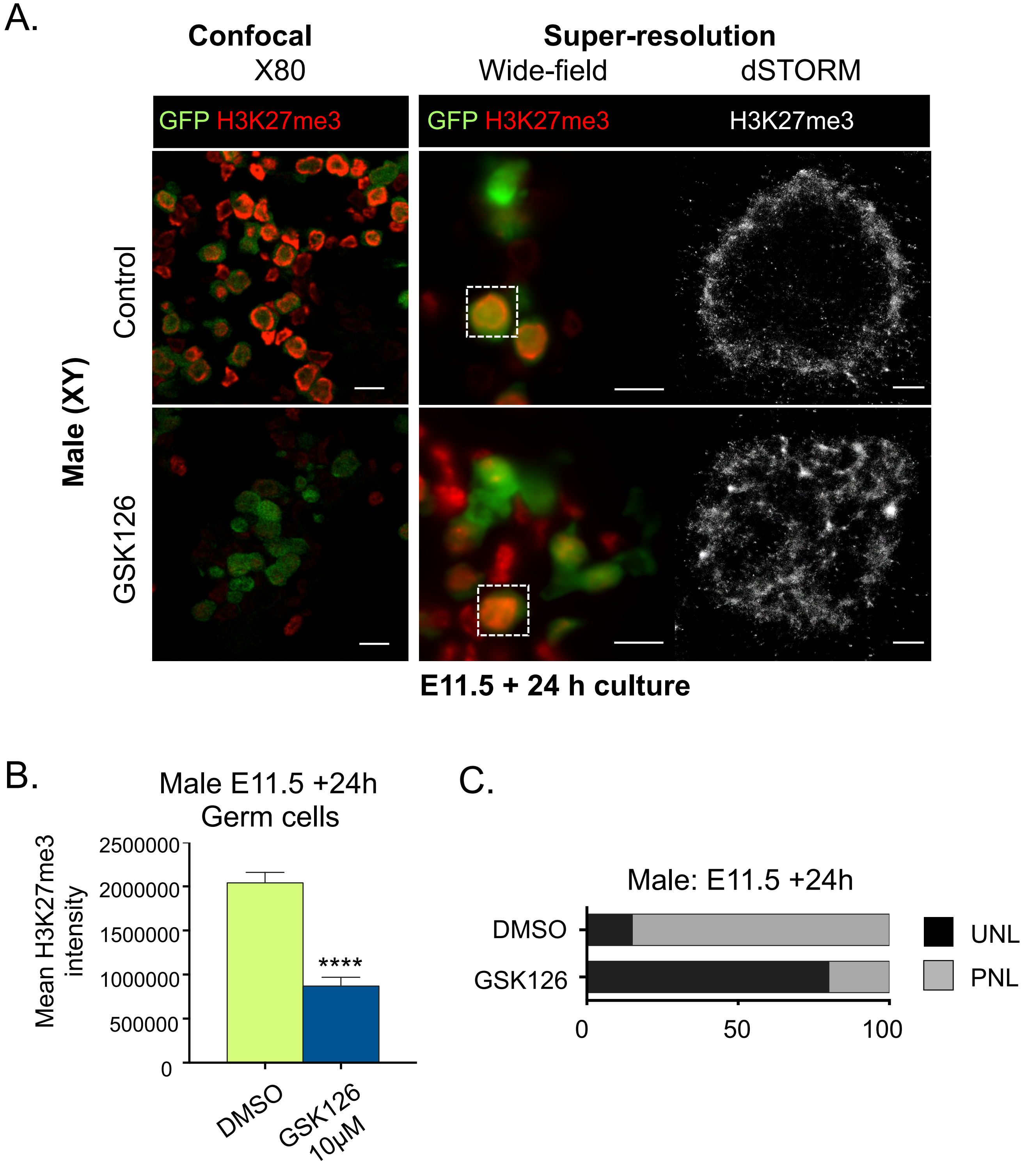

Prokopuk *et al* Additional file 1: Fig. S4

Fig. S5: Blockage of EZH1/2 significantly reduces H3K27me3 enrichment near the nuclear lamina in fetal germ cells undergoing epigenetic reprogramming. Radial histogram quantification of dSTORM super-resolution immunofluorescent images of germ cells in sections from XY E11.5 fetal gonad/mesonephros tissues cultured for 24h**.** E11.5 wildtype control is shown in green, E11.5+24h vehicle control (DMSO) is shown in blue, and E11.5+24h of GSK126 treatment (10μM) shown in red. Left image of each colored panel (160x) represents merged channels, with green marking germ cells (eGFP) and H3K27me3 shown in the red channel. 10μm scale bars. The right-hand image of each panel shows dSTORM super-resolution images of germ cells (H3K27me3 in greyscale). 1μm scale bars. 3-6 super-resolved images from three biological replicates. Y-axis shows relative H3K27me3 intensity and X-axis shows radial distance (μm) from the center of the nucleus. Error bars ± SEM.

Male E11.5 + 24h

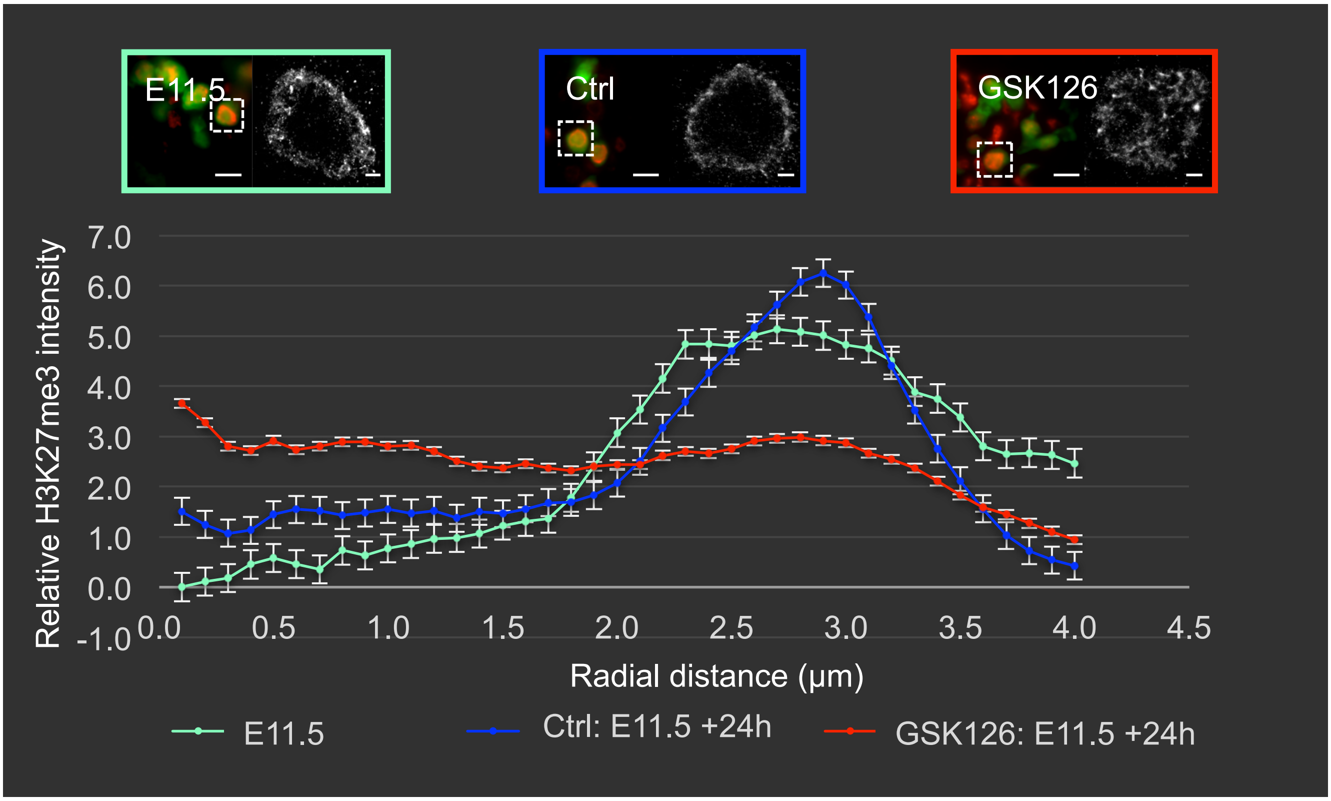

Prokopuk *et al* Additional file 1: Fig. S5

Table S1: Tissue Fixation times (4% PFA in 1 x PBS)

Time Point	Fixing time (4% PFA in PBS)
E10.5 (embryo mid-section)	40 minutes, room temperature
E11.5 gonads (embryo mid-section)	25 minutes, room temperature
E12.5 gonads	20 minutes, room temperature
E13.5 gonads	40 minutes, room temperature
E15.5 gonads	90 minutes, room temperature
Postnatal day gonads	Overnight at 4°C

Table S2: Primary Antibodies

**Antibody**	**Species**	**Dilution (IF)**	**Dilution (flow cytometry)**	**Supplier, catalogue number**
**EED**	Sheep	1:100	-	R & D, AF5827
**EZH2**	Rabbit	1:400	-	Cell Signalling Technologies, D2C9
**H3K27me3**	Rabbit	1:400	1:40	Cell Signalling Technologies, C36B11
**SUZ12**	Rabbit	1:100	-	Cell Signalling Technologies, D39F6
**IgG**	Rabbit	-	1:250	Cell Signalling Technologies, DA1E

Table S3: Secondary Antibodies

**Antibody**	**Species**	**Dilution**	**Supplier, catalogue number**
Sheep 647	Donkey	1:300	Alex Fluor (Life Technologies), A21447
Rabbit 594	Donkey	1:300	Alex Fluor (Life Technologies), A21207
Goat 647	Donkey	1:300	Alex Fluor (Life Technologies), A21447
Rabbit 647	Donkey	1:300	Alex Fluor (Life Technologies), A21447
